# Human Beta-Defensin 2 and 3 Inhibit HIV-1 Replication in Macrophages

**DOI:** 10.3389/fcimb.2021.535352

**Published:** 2021-07-01

**Authors:** Jennifer P. Bharucha, Lingling Sun, Wuyuan Lu, Suzanne Gartner, Alfredo Garzino-Demo

**Affiliations:** ^1^ Division of Virology, Pathogenesis, and Cancer, Institute of Human Virology, University of Maryland School of Medicine, Baltimore, MD, United States; ^2^ Department of Biochemistry and Molecular Biology, University of Maryland School of Medicine, Baltimore, MD, United States; ^3^ Department of Medicine, University of Maryland School of Medicine, Baltimore, MD, United States; ^4^ Department of Microbiology and Immunology, University of Maryland School of Medicine, Baltimore, MD, United States; ^5^ Department of Molecular Medicine, University of Padova, Padova, Italy

**Keywords:** macrophages, HIV-1, human β-defensin 2, CCRs, APOBEC3G, APOBEC3A

## Abstract

Human beta-defensins (hBDs) are broad-spectrum antimicrobial peptides, secreted by epithelial cells of the skin and mucosae, and astrocytes, which we and others have shown to inhibit HIV-1 in primary CD4^+^ T cells. Although loss of CD4^+^ T cells contributes to mucosal immune dysfunction, macrophages are a major source of persistence and spread of HIV and also contribute to the development of various HIV-associated complications. We hypothesized that, besides T cells, hBDs could protect macrophages from HIV. Our data in primary human monocyte-derived macrophages (MDM) *in vitro* show that hBD2 and hBD3 inhibit HIV replication in a dose-dependent manner. We determined that hBD2 neither alters surface expression of HIV receptors nor induces expression of anti-HIV cytokines or beta-chemokines in MDM. Studies using a G-protein signaling antagonist in a single-cycle reporter virus system showed that hBD2 suppresses HIV at an early post-entry stage *via* G-protein coupled receptor (GPCR)-mediated signaling. We find that MDM express the shared chemokine-hBD receptors CCR2 and CCR6, albeit at variable levels among donors. However, cell surface expression analyses show that neither of these receptors is necessary for hBD2-mediated HIV inhibition, suggesting that hBD2 can signal *via* additional receptor(s). Our data also illustrate that hBD2 treatment was associated with increased expression of APOBEC3A and 3G antiretroviral restriction factors in MDM. These findings suggest that hBD2 inhibits HIV in MDM *via* more than one CCR thus adding to the potential of using β-defensins in preventive and therapeutic approaches.

## Introduction

Mucosal surfaces, especially of the genital and gastrointestinal tracts, are the primary sites of initial transmission of HIV-1. The virus can reach target cells across both intact and damaged mucosal surfaces *via* different mechanisms [reviewed in (Moutsopoulos et al., 2006)]. Virus may also enter *via* damaged mucosal surfaces to infect susceptible dendritic cells (DCs), macrophages and T cells. Regardless of the mode of entry, once the virus has breached the mucosal barrier and entered susceptible target cells, including macrophages, it is subsequently transported *via* the lymphatic system and blood stream to other sites in the body.

Macrophages are versatile cells of the immune system. They can independently recognize and attack foreign antigens, activate various aspects of the innate immune response, as well as interact with and activate cells of the adaptive immune response (Dobrovolskaia and Vogel, 2002; Mantovani et al., 2004; Gordon and Taylor, 2005; Gordon and Mantovani, 2011; Sica and Mantovani, 2012; Wynn et al., 2013). Macrophages are susceptible to infection by HIV and are in mucosae, potentially exposing them to infection during heterosexual transmission (Greenhead et al., 2000). Several studies have shown that cells of the monocyte/macrophage lineage serve as both, an active site for virus replication and dissemination through the body (Gartner et al., 1986b), especially to protected sites such as perivascular macrophages (Williams et al., 2001) and microglia in the central nervous system (Gartner et al., 1986a; Koenig et al., 1986), and as a reservoir of latent virus (Gendelman et al., 1989; Brown et al., 2006; Li et al., 2010; Honeycutt et al., 2016; Honeycutt et al., 2017; Ganor et al., 2019; Ko et al., 2019). Additionally, infected macrophages alter the innate immune response, making the host more vulnerable to other infections. Thus, by virtue of their importance in the regulation of the immune response, their relatively long life span, and their susceptibility to infection, macrophages contribute to the persistence and amplification of HIV infection [reviewed in (Alexaki et al., 2008; Koppensteiner et al., 2012; Churchill and Nath, 2013)].

Current antiretroviral therapy (ART) for HIV infection has evolved tremendously over the past thirty years and has resulted in significant reductions in morbidity and mortality. Despite these advances, toxicity, multi-drug resistance, lack of response to drugs, failure to restore immune competence and to eradicate latent virus reservoirs are some of the common problems associated with ART. The problem is further compounded by the high cost, lack of compliance, and/or unavailability of treatment and patients also remain susceptible to the serious complications of AIDS. In particular, while the introduction of cART has significantly decreased the occurrence of HIV-associated dementia, and the incidence of AIDS, the prevalence of HIV-Associated Neurocognitive Disorders (HAND) has increased despite long standing viremia suppression [reviewed in (Broder, 2010; Deeks, 2013; Nath and Tyler, 2013)]. Hence, there is an urgent need to develop strategies that serve as complementary or alternative therapies.

Components of innate immunity participate to control HIV infection and studying their mechanisms of action may contribute to the development of new treatments. Our studies highlight potential therapeutic application of human defensins and the pathways that they induce in cells susceptible to HIV infection.

Defensins are a heterogeneous group of small molecular weight peptides that exhibit potent antimicrobial properties against a broad variety of pathogens, including bacteria, fungi, and viruses [reviewed in (Lehrer and Lu, 2012; Jarczak et al., 2013; Wilson et al., 2013; Holly et al., 2017; Brice and Diamond, 2020)]. In addition, they are involved in stimulation, proliferation, differentiation, morphogenesis, motility and function of immune cells, hence, playing significant roles in both innate and adaptive immunity [reviewed in (Yang et al., 2007)]. Several studies have also identified defensins as potential immunotherapies for different cancers (Papo and Shai, 2005; Kesting et al., 2012; Mei et al., 2012), auto-immune (Badr, 2013) and inflammatory disorders (Niyonsaba et al., 2001; Poiraud et al., 2012). In humans, two major subclasses, α- and ß-defensins, are produced. Human β-defensins are produced primarily by epithelial cells of diverse mucosal tissues and by monocytes, macrophages, astrocytes and DCs [reviewed in (Yang et al., 2007)]. Immunohistochemical studies show that hBD2 is constitutively expressed in the oral mucosa of normal healthy individuals, producing a barrier across the epithelium. In contrast, a previous study from our group showed that hBD2 levels were not detectable in HIV-1-positive individuals (Sun et al., 2005), which may predispose them to oral complications of AIDS.

Studies by various groups over the years show that both α- and ß-defensins exhibit anti-HIV activity *in vitro*. α-defensins can inhibit HIV-1 replication by direct interaction and inactivation of the virions or by affecting target cells (Nakashima et al., 1993; Mackewicz et al., 2003; Wang et al., 2004; Chang et al., 2005). Previous studies from our laboratory (Sun et al., 2005; Lafferty et al., 2010; Lafferty et al., 2017) and from others (Quinones-Mateu et al., 2003; Feng et al., 2006) show that hBD2 and hBD3 elicit anti-HIV activity in peripheral blood mononuclear cells (PBMCs) and CD4^+^ T cells. It was further shown by our group (Lafferty et al., 2010; Lafferty et al., 2017) that hBD2 inhibits HIV at an early stage post-entry and the intracellular mechanism involves induction of the host anti-viral restriction factor apolipoprotein B mRNA-editing enzyme-catalytic polypeptide-like 3G (APOBEC3G) *via* the CC chemokine receptor 6 (CCR6). CCR6 is expressed on cells that are highly permissive to HIV infection, including memory T cells, Th_17_ cells, α4ß7 cells, and microglia (Liao et al., 1999; Flynn et al., 2003; Acosta-Rodriguez et al., 2007; Singh et al., 2008; El Hed et al., 2010; Gosselin et al., 2010; Monteiro et al., 2011; Christensen-Quick et al., 2016; Lafferty et al., 2017). It has been shown that chemokine receptors can be functionally bound by non-chemokine ligands such as defensins (Yang et al., 1999; Jin et al., 2010; Lafferty et al., 2010; Rohrl et al., 2010).

Due to the lack of studies on macrophages, we tested the ability of hBD2 to inhibit HIV-1 infection in primary human monocyte-derived macrophages (MDM) and elucidate its mechanism(s) of action. We demonstrate that in MDM, hBD2 inhibits HIV post-entry *via* more than one mechanism. It inhibits virus at an early stage in the life cycle *via* binding to and signaling through more than one CCR type that results in induction of anti-retroviral restriction factors of the APOBEC3 family i.e. 3G and 3A. This suggests that the mechanism(s) of hBD2-mediated HIV-1 inhibition in primary human macrophages is quite different from that in primary human CD4^+^ T cells.

## Materials and Methods

### Ethics Statement

Human PBMCs were isolated from healthy blood donor’s leukopaks obtained from New York Blood Center, Long Island, NY, in accordance with their guidelines. Donors were anonymous; hence, patient consent was not required.

### Reagents

Recombinant human IFN-α was obtained from R&D Systems, Inc. The HIV-1 reverse transcriptase (RT) inhibitor azidothymidine (AZT); the chemical antagonist of CCR2, RS102895, Cytochalasin D (CytD) and paraformaldehyde were purchased from Sigma-Aldrich. Pertussis toxin (Bordetella pertussis, glycerol solution) was from Calbiochem. TURBO DNase I was from Ambion. The HIV-1 fusion inhibitor T20 peptide was a kind gift from Dr. Lai-Xi Wang at the Institute of Human Virology.

### Isolation and Culture of Primary Cells

Human PBMCs were isolated from leukopaks from healthy human subjects with the use of Histopaque-1077 (Sigma-Aldrich). Monocyte-derived macrophages (MDM) were prepared by adherence method. Cells were plated at ~2x10^6^ cells/ml in 100mm petri dishes (Corning) and left to differentiate for 5–7 days in Roswell Park Memorial Institute (RPMI)-1640 (Cellgro, Mediatech, Inc.) complete medium (which is supplemented with 1% penicillin/streptomycin, 2 mM L-glutamine [Quality Biochemical, Gaithersburg, MD], 20% heat-inactivated Fetal Bovine Serum [Gemini Bio-Products]) in the presence of 10% human AB serum (Gemini Bio-Products). Non-adherent cells were removed by thorough washing and differentiated macrophages were cultured in RPMI-1640 complete medium only (no human serum here onwards). In this study, differentiated macrophages were detached with StemPro Accutase (GIBCO by Life Technologies) following manufacturer’s recommendations and gentle scraping when needed. Flow cytometry analyses confirmed that more than 95% of the adherent cells were macrophages. Cell viability was determined using trypan blue exclusion method.

### Virus Production

HIV-1_BaL_ (R5 isolate) virus stocks were prepared in MDM, while the transmitted-founder isolate HIV-1AD17 (kindly provided by George Shaw was produced in PM1 cells (Li et al., 2010). Virus p24 levels were measured using a commercial ELISA kit (Perkin Elmer, Foster City, CA). TCID_50_ of virus stocks were determined in PBMCs using the protocol of ACTG Laboratory Technologist Committee. Luciferase reporter pseudotyped virus was generated by cotransfection of 293T cells with pNL4-3.Luc.R^-^E^-^ plasmid which has the firefly luciferase gene inserted into the *nef* gene, and an Amphotropic Murine Leukemia Virus (AMLV) envelope-expressing plasmid, using Fugene 6 (Roche Diagnostics and Promega) and plasmids kindly provided by Dan R. Littman (New York University) (Page et al., 1990). Supernatants from 293T cells were harvested 72 hours after cotransfection and p24 levels measured as described above. Virus was concentrated, if needed, by ultra-centrifugation on a sucrose cushion. Virus was titrated in primary MDM.

### Total Chemical Synthesis of Human β-Defensins

hBDs 1, 2 and 3 were chemically synthesized by solid phase peptide synthesis with a custom-modified procedure tailored from the published *in situ* neutralization protocol developed for Boc chemistry (Schnolzer et al., 1992). The syntheses, purification, folding, and characterizations were published previously (Wu et al., 2003). The beta connectivity of three disulfide bonds (Cys1-Cys5, Cys2-Cys4, and Cys3-Cys6) in highly pure synthetic hBDs 1 to 3 was independently verified by mass mapping of peptide fragments generated by enzymatic digestion and Edman degradation (Wu et al., 2003). Protein concentrations were determined by absorbance measurements at 280 nm using molar extinction coefficients calculated according to a published algorithm (Pace et al., 1995).

### Cell Metabolism Assay

Cell metabolism was determined by using the MTS [3,4-(5-dimethylthiazol-2-yl)-5-(3-carboxymethoxy phenyl)-2-(4-sulfophenyl)-2H-tetrazolium salt] assay (Promega, Madison, WI), which measures conversion of MTS tetrazolium into formazan by cellular dehydrogenase enzymes in metabolically active cells. For this, MDM (10^5^ cells/well) were cultured in triplicate in 96-well plates with media alone as a control or hBDs for 3-4 days and then the MTS/PMS mixture was added as per manufacturer’s protocol and incubated for 1 to 4 hours before spectrophotometric absorbance reading at 490 nm. Triplicate readings were averaged and OD ratios of treated/control cells were calculated as percentages.

### Infectivity Assays

MDM (2x10^6^ cells/ml) were infected with 6-8x10^4^ TCID_50_/ml of HIV-1_BaL_ for 2 hours followed by three washes, and then cultured at 10^5^ cells/well of 96-well plate in RPMI complete medium with the appropriate treatment(s) up to 14 days at 37°C. Half of the culture supernatants were changed with fresh medium on days 4, 7 and 10. Each culture was performed in triplicate. As a positive control, cells were pretreated with AZT (10 µM) prior to infection. Infection/HIV-1 replication was determined by measuring the amount of p24 antigen in the culture supernatants using a commercially available p24 antigen capture assay kit (Perkin Elmer, Foster City, CA) following manufacturer’s protocol. For experiments with the chemical antagonist of CCR2, RS102895, after infection of MDM with HIV-1BaL, cells were pretreated with the inhibitor (15 µM) at 37°C for 2 hours, followed by addition of hBD2, and cultured as described above. Percentage of inhibition in treated cells was calculated with the use of the following formula: % Inhibition= [1 − (p24_treated_/p24_control_)] x 100, where p24_treated_ and p24_control_ are concentrations of HIV-1 p24 measured in supernatants of treated and untreated cells, respectively.

Single-round infections were performed with the use of AMLV envelope-pseudotyped HIV-1. MDM were incubated with pseudotyped virus for 3 hours and subsequently washed with PBS and then cultured (2x10^5^ cells/well of 48-well plate) in RPMI complete medium in the presence or absence of hBD2 (4.7 µM) for 3 days at 37°C. Following this, infected cells were washed with PBS and lysed with Reporter Lysis Buffer (SteadyGlo kit, Promega), and luciferase activity was measured in a Turner Luminometer. For inhibition of G_i_-protein signaling with pertussis toxin (PTx), cells were infected with pseudotyped virus as described above followed by treatment with PTx (100 ng/ml) for at least 3 hours and then cultured in the presence or absence of hBD2.

### Flow Cytometry

Effect of hBD2 on cell surface expression of HIV-1 receptor and co-receptors, CD4, CCR5 and CXCR4 was analyzed by flow cytometry. For this, MDM were treated with hBD2 and hBD3 (4.7 µM) for time periods described in the *Results* section below, after which the cells were harvested, processed and stained. Briefly, the cells were washed with cold FACS buffer (PBS containing 2% FBS and 0.1% Na-azide), and then blocked with either 5% human AB serum or human FcR blocking reagent (Miltenyi Biotec) in FACS buffer for up to 30 minutes followed by washing and staining with mouse monoclonal anti-human antibodies (mAbs) for 30 minutes at 4°C in the dark. After incubation, cells were washed and fixed in 1% paraformaldehyde in FACS buffer. The cell preparations were analyzed with a FACS Calibur flow cytometer (BD Biosciences, CA). Macrophage surface markers including CD14, CD11b, CD36, and HLA-DR were used to identify and gate on macrophages. Live cells were gated to exclude all nonviable cells and debris according to forward and scatter profiles. These gated events were further analyzed for expression of CD4, CCR5 and CXCR4. Positives and negatives were determined by comparison with matching isotype controls. The following mAbs were used: PE-conjugated anti-CD4, APC-conjugated anti-CCR5, and PE-conjugated anti-CXCR4 (all from BD Pharmingen, San Jose, CA). In all cases, isotype-matched control mAbs were used. For flow cytometry analysis of cell surface expression of CCR2 and CCR6 on MDM, similar FACS staining method was used with following mAbs: APC-conjugated anti-CCR2 (R&D Systems, Inc.) and PE-conjugated anti-CCR6 (BD Pharmingen). All data were analyzed using FlowJo software (Tree Star Inc., San Carlos, CA).

### Quantitation of Interferons and β-Chemokines in hBD2 Treated MDM Supernatants

Supernatants derived from MDM incubated for different times up to 24 hours with hBD2 (4.7 µM) were tested for the presence of interferon-α and –β and the β-chemokines RANTES, MIP-1α and MIP-1β by commercial ELISA kits following the manufacturer’s protocols (R&D Systems, Inc.).

### Real-Time Quantitative PCR of HIV-1 DNA

MDM (10^6^ cells/time point) were untreated or pretreated with AZT (10 µM) or fusion inhibitor T20 (2.5 µM) and infected for 2 hours at 37°C with 10^5^ TCID_50_/ml of DNase I–treated HIV-1_BaL_. Cells were washed to remove extracellular virus, and resuspended in RPMI complete media, and infected untreated cells were incubated in the presence or absence of hBD2 (4.7 µM). Total cellular DNA was extracted with the use of the DNeasy Blood and Tissue Kit (QIAGEN) at 6, 12, 24 and 48 hours post-infection. DNA was analyzed by real-time quantitative PCR to determine the number copies of early (negative strand strong stop) reverse transcripts present with iQSYBR green supermix (Bio-Rad) and primers F: 5’-GGCTAACTAGGGAACCCACTG-3’ and R: 5’-CTGCTAGAGATTTTCCACACTGAC-3’ (Lafferty et al., 2010). Transcript levels were normalized using endogenous *albumin* gene as a reference. Primers used for *albumin* are F: 5’-TGTTGCATGAGAAAACGCCA -3’ and R: 5’-GTCGCCTGTTCACCAAGGAT -3’ (Lafferty et al., 2010). Annealing temperatures of 60°C and 62°C were used for strong stop and *albumin*, respectively. Reactions were performed in triplicate with the use of a Bio-Rad iQ5 Real-Time PCR machine. A standard curve for number of HIV-1 DNA copies was set up with dilutions of HxB2 plasmid DNA. Data was analyzed with Bio-Rad iQ5 and Microsoft Excel software.

### Real-Time Quantitative RT-PCR Analysis of APOBEC3G and APOBEC3A

Cells (10^6^ cells per time point) were untreated or treated with hBD2 (4.7 µM) or IFN-α (1000 U/ml). RNA was extracted with the Rneasy Kit (Qiagen) at the indicated time points. First strand cDNA was synthesized from 500 ng total RNA with iScript cDNA Synthesis Kit (Bio-Rad). cDNA was analyzed by real-time quantitative PCR with iQSYBR green supermix (Bio-Rad) with the use of primers specific for APOBEC3G; F: 5’-CGCAGCCTGTGTCAGAAAAG-3’ and R: 5’-CCAACAGTGCTGAAATTCGTCATA-3’ (Jin et al., 2005), and for 18S ribosomal RNA; F: 5’-ATCAACTTTCGATGGTAGTCG-3’ and R: 5’-TCCTTGGATGTGGTAGCCG-3’ (Lafferty et al., 2010) and annealing temperature of 60°C. For APOBEC3A, cDNA was synthesized as described above and analyzed by real-time quantitative PCR with iQ Supermix (Bio-Rad) and Taqman primer-probe sets Hs00377444_m1 APOBEC3A-FAM and Hs03928985_g1 RN18S1-VIC_PL (Applied Biosystems) and annealing temperature of 60°C. In both cases, all reactions were performed in triplicate with the use of a Bio-Rad iQ5 Real-Time PCR machine. Data was analyzed with Bio-Rad iQ5 and Microsoft Excel software. The ΔΔCt method was used to calculate fold change between untreated and treated cells normalized to 18S ribosomal RNA.

### Immunoblotting

For CCR6 western blots, untreated MDM and JKT-FT7 cells were lysed with RIPA buffer (Sigma) containing 0.1 mM PMSF, 1X EDTA-free protease inhibitor cocktail (Sigma). Total protein concentration was determined with the BCA Protein Assay kit (Pierce, Thermo Scientific, Inc.), and equal amounts of total protein were subjected to sodium dodecyl sulfate–polyacrylamide gel electrophoresis analysis. Immunoblots were probed with either mouse monoclonal anti-human CCR6 antibody (MAB195, R&D Systems, Inc.) or rabbit polyclonal anti-human CCR6 antibody (ab78429, Abcam Inc.) followed by horseradish peroxidase-conjugated anti-mouse or anti-rabbit secondary antibodies (Santa Cruz Biotechnology) detected with the ECL-plus kit (GE Healthcare, Bucks, UK) on a PharosFX Plus Molecular Imager (Bio-Rad). Immunoblotting for CCR6 in CD4^+^ T CCR6^+^ and CCR6^-^ sorted cell lysates was performed on a separate blot that was previously used for another experiment in our lab. The low frequency of these subsets of cells in total human PBMCs and the high cost involved limits the availability of whole cell lysates from these populations.

For APOBEC3 induction experiments, MDM were incubated in the presence or absence of increasing concentrations of hBD2 or IFN-α (1000 U/ml) for 0, 4, 8, and 24 hours. Cells were lysed as described above. Immunoblots were probed with either rabbit polyclonal anti-hAPOBEC3G antisera or rabbit anti-hAPOBEC3G-C antisera for APOBEC3A and mouse anti-hβ-actin antibody (Abcam Inc.) or rabbit anti-GAPDH monoclonal antibody (Cell Signaling Technology, Inc.) as load control followed by horseradish peroxidase-conjugated anti-rabbit or anti-mouse secondary antibodies (Santa Cruz Biotechnology) detected with the ECL-plus kit (GE Healthcare, Bucks, UK). The polyclonal anti-APOBEC3G antibodies from Drs. Warner C. Greene (#9968), Klaus Strebel (anti-ApoC17 #10082), and Jaisri Lingappa (#10201) and, rabbit anti-APOBEC3G-C antibody from Dr. Klaus Strebel (#9906) (Kao et al., 2003) used to detect APOBEC3A were obtained through the National Institutes of Health AIDS Research and Reference Reagent Program, Division of AIDS, National Institute of Allergy and Infectious Diseases, National Institutes of Health. Densitometric quantification of protein levels was done with Quantity One software, version 4.6.9 (Bio-Rad Laboratories, Inc., CA, USA). Each lane/sample was normalized to its respective load control, and background signal for the blot was subtracted from all lanes.

### Replicates

With the exception of FACS analyses and pseudotyped HIV infections, every experiment was performed in triplicate with cells from a particular donor and was performed independently in cells from 3 or more donors. The number of donors used is noted in each figure legend as *n*.

Data analyses was performed using Microsoft Excel and GraphPad Prism 5. Images and figures were prepared using Adobe Photoshop 7.0 or CS software (Adobe Systems, San Jose, CA).

## Results

### hBD2 Inhibits HIV-1 Replication in Macrophages in a Dose-Dependent Manner

**Figure 1:**
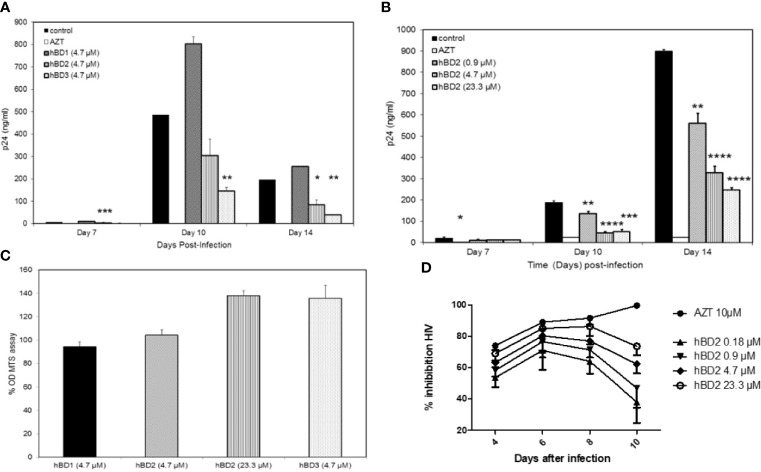
Human β-defensins inhibit HIV-1 replication in macrophages *in vitro*. MDM were infected with HIV-1_BaL_. After virus removal and washing, hBD1, hBD2, and hBD3 (4.7 µM) **(A)** or increasing concentrations of hBD2 (0-23.3 µM) **(B)** were added to cultures. Cells were pretreated with AZT as control. Infection was monitored by assaying supernatants for HIV p24 production by ELISA at the times indicated. Data are presented as mean ± SEM of triplicates. Representative experiment, *n*=3. *P < 0.05, **P < 0.005, ***P < 0.0005, ****P < 0.0001 between treatment and control groups determined with unpaired two-tailed *t* test. **(C)** ß-defensins are not toxic to macrophages at the concentrations that inhibit HIV-1. Cells treated with hBDs were tested using MTS assay. Cells were cultured in triplicate in 96-well plates for 3 days in the presence or absence of β-defensins; MTS mix was added and incubated 1 to 4 hrs prior to spectrophotometric absorbance readings at 490 nm. Triplicate readings were averaged (± SEM) and percentage OD ratios of treated/control cells were calculated. **(D)** hBD2 inhibit infection of MDM with a transmitted-founder HIV strain. MDM were infected with transmitter-founder virus AD17. After virus removal and washing, hBD2 at concentrations indicated above were added to cultures. HIV p24 release in supernatants was monitored by ELISA at the time indicated, and % inhibition was calculated as % of HIV p24 production from untreated MDM. Data are presented as mean ± SEM of triplicates, N=3.

To evaluate the effect of hBDs 1, 2 and 3 on replication of R5 virus in primary human MDM, cells were infected with 1.24x10^3^ TCID_50_ of HIV-1_BaL_ and then cultured in the absence or presence of hBD 1, 2 or 3, at a final concentration of 4.7 µM, for up to 14 days. AZT was used as a positive control in all infection experiments. Release of HIV-1 p24 was quantified in supernatants every 3 days starting at day 7 after infection. **Figure 1A** shows p24 amounts in the culture supernatants. Both hBD2 (40-57%) and hBD3 (70-80%) inhibited HIV-1 replication, in contrast to hBD1 which, at the concentration tested, appears to increase HIV-1 replication in MDM. To test for toxicity of hBDs on MDM, cell metabolism was measured by the MTS assay on cells treated with hBD 1, 2 or 3 for 3 to 4 days. As shown in **Figure 1C**, at the concentrations used, these ß-defensins have a marginal positive effect on cell metabolism in MDM. Although hBD3 shows more potent inhibition of HIV-1 infection in macrophages as compared to hBD2 and it does not appear to be affecting cell metabolism, it was shown to decrease proliferation of PBMCs (Sun et al., 2005), thereby making it a less desirable candidate as compared to hBD2 for further studies. Hence, from here onward, studies to determine and characterize the mechanism(s) of ß-defensin-mediated suppression of HIV-1 infection in macrophages were limited to hBD2. To evaluate the effect of different concentrations of hBD2 on HIV infection in MDM, cells were infected and cultured as described above in the presence or absence of hBD2 concentrations ranging from 0.9 to 23.3 µM (corresponds to 4 to 100 µg/ml). As shown in **Figure 1B**, hBD2 significantly inhibited R5 virus replication in a dose-dependent manner. We also tested the effect of hBD2 in a transmitted/founder virus, AD17 (Li et al., 2010). As seen with BaL, 4.7 µM concentration shows inhibition ranging from 45-65% over time, and the higher concentration of 23.3 µM shows inhibition ranging from 70-80% over time (**Figure 1D**). These results, taken together with published reports in PBMCs and CD4^+^T cells (Quinones-Mateu et al., 2003; Sun et al., 2005), suggest that the effect of hBD2 on HIV-1 replication extends to macrophages. Since the concentration of 4.7 µM hBD2 is well within the range of secreted hBD2 measured in oral mucosa and epidermal tissues (Liu et al., 2002), we used this concentration in further studies.

### hBD2 Does Not Alter Surface Expression of HIV Receptors on Macrophages

**Figure 2:**
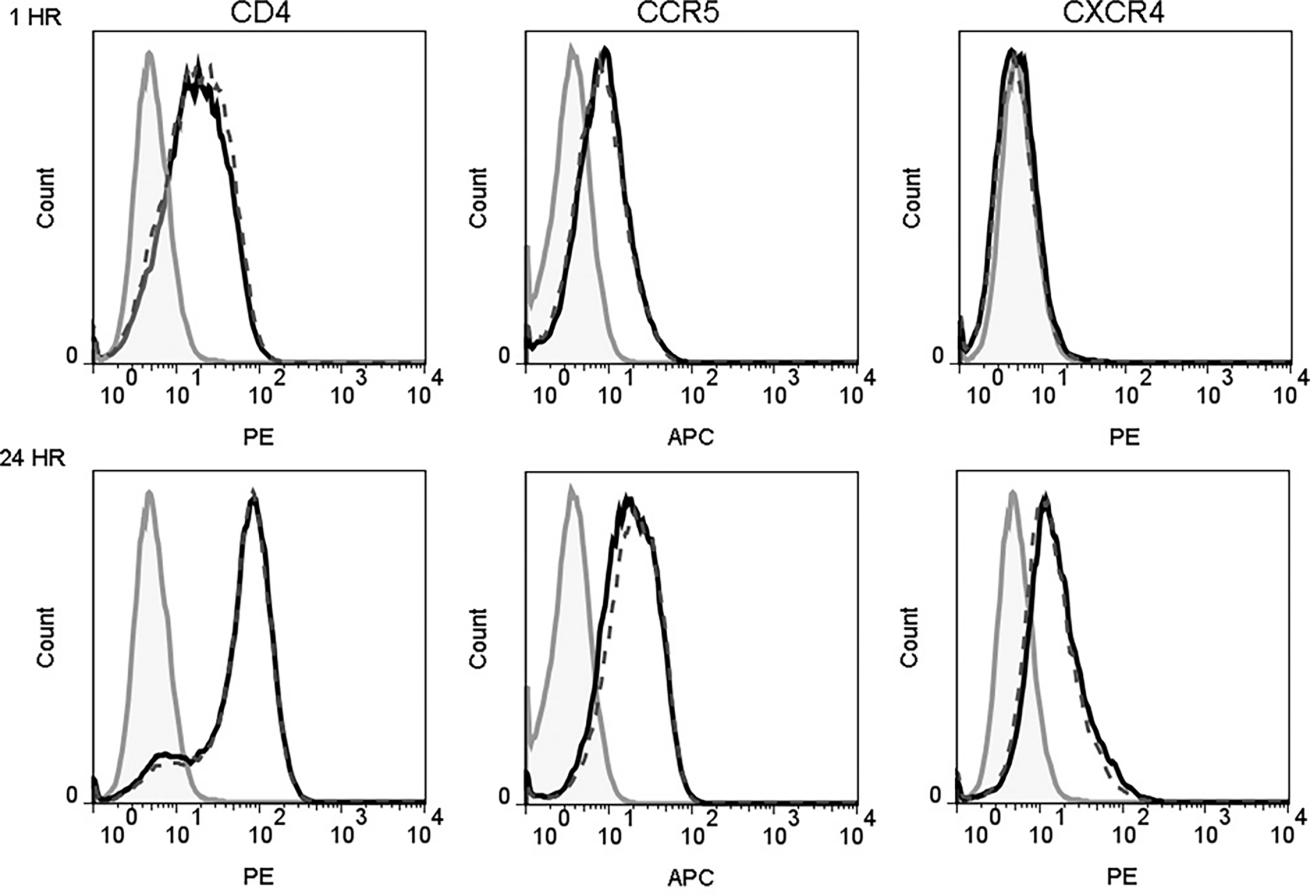
hBD2 does not alter surface expression of HIV receptors on macrophages. MDM were cultured in the absence (black solid lines) or presence (black dotted lines) of hBD2 for different times. The surface expression of CD4, CCR5, and CXCR4 was assessed by flow cytometry as described in *Materials and Methods*. Data analyzed using FlowJo software. Left, middle, and right panels show staining of CD4, CCR5, and CXCR4, respectively. Isotype-matched control antibodies are shown in grey. The x-axis and y-axis show fluorescence intensity and cell count, respectively. Representative experiment, *n*=2.

Previous studies have shown that β-defensins alter HIV-1 coreceptor CXCR4 expression on PBMCs and CD4^+^ T cell lines (Quinones-Mateu et al., 2003; Feng et al., 2006). This raises the possibility that interaction of hBD2 with macrophages may affect the expression pattern of HIV-1 receptor and coreceptors. To test this hypothesis, we measured cell surface expression of CD4, CCR5 and CXCR4 on cells after treatment with hBD2 for different time periods. To this end, we treated uninfected MDM with hBD2 for 1, 2, 3, and 24 hours followed by staining with PE-CD4 or PerCP-CD4, APC-CCR5, PE-CXCR4, and isotype-matched control antibodies and flow cytometric analyses. hBD2 had no significant effect on the surface expression of CD4, CXCR4 or CCR5 on MDM. **Figure 2** shows the results at 1 and 24 hours post-treatment with hBD2; similar results were obtained with 2 and 3 hours of treatment (data not shown). **Table 1** shows the median fluorescence intensity (MFI) values for each surface marker at the different times tested. These results indicate that treatment with hBD2 does not modulate HIV receptors. We observed similar results treating MDMs with hBD3 (**Figure S1**).

**Table 1:** Median Fluorescence Intensity (MFI) values for surface receptor expression on MDM.

Time (hr) post treatment	Condition	Donor 1			Donor 2		
		CD4-PE	CCR5-APC	CXCR4-PE	CD4-PE	CCR5-APC	CXCR4-PE
1	untreated	17.30	8.27	4.71	11.35	6.82	2.97
	hBD2	17.72	7.77	4.58	10.76	6.53	2.83
2	untreated	19.04	8.82	5.25	11.29	7.35	3.28
	hBD2	20.07	9.12	5.00	11.17	7.27	2.94
3	untreated	21.89	9.60	5.34	13.67	8.42	3.39
	hBD2	21.61	9.53	5.12	12.53	8.67	3.32
24	untreated	68.72	18.12	14.04	45.86	14.40	5.50
	hBD2	70.00	20.26	12.51	48.33	20.86	5.57

### hBD2 Does Not Reproducibly Induce Expression of Anti-Viral Cytokines or β-Chemokines in Macrophages

Type I interferons (IFN) -α and -ß are induced in response to viral infections and are the cytokines that suppress HIV replication both *in vitro* and *in vivo* [reviewed in (Shankar et al., 2012)]. Several studies have also demonstrated the significance of ß-chemokines in restriction of, and protection from, HIV infection both *in vitro* and *in vivo* [reviewed in (Garzino-Demo et al., 2000; DeVico and Gallo, 2004)]. Previous studies have shown that α-defensins can inhibit HIV-1 replication in macrophages by triggering release of HIV-1 inhibitory ß-chemokines (Guo et al., 2004) and that treatment of DCs with hBD2 upregulated the expression and release of ß-chemokines (MIP-1α and MIP-1ß) in culture supernatants (Biragyn et al., 2002). Therefore, we tested whether hBD2 inhibition of HIV-1 in MDM is mediated *via* up-regulation of the expression of (anti-R5 tropic) ß-chemokines- MIP-1α, MIP-1ß and RANTES and/or Type I IFNs. Culture supernatants obtained from MDM treated with hBD2 up to 24 hours were used to quantify these molecules by commercial ELISA kits. As shown in **Figure S2**, treatment with hBD-2 induced an increase in production of all three ß-chemokines at the 24 hour time point in Donor 1, and an increase in production of RANTES at the 4 and 8 hour time points in Donor 3 as compared to levels in untreated cultures. It is unlikely; however, that the modest increase in β-chemokines observed at different time points post-treatment significantly contribute to the anti-HIV-1 activity of hBD2 in macrophages as the concentrations measured in the hBD2 treated culture supernatants are well below the concentrations needed to efficiently inhibit HIV replication (Cocchi et al., 1995). Boniotto et al. had made similar observations with hBD2 in PBMCs (Boniotto et al., 2006). Further, IFN-α and -ß were not detected in supernatants of both untreated control cells as well as hBD2 treated cells at any time points in any of the donors (data not shown). These data suggest that the anti-HIV-1 effect of hBD2 in macrophages is not mediated through induction and release of these anti-viral cytokines and ß-chemokines.

### hBD2 Suppresses HIV-1 at an Early Post-Entry Stage

**Figure 3:**
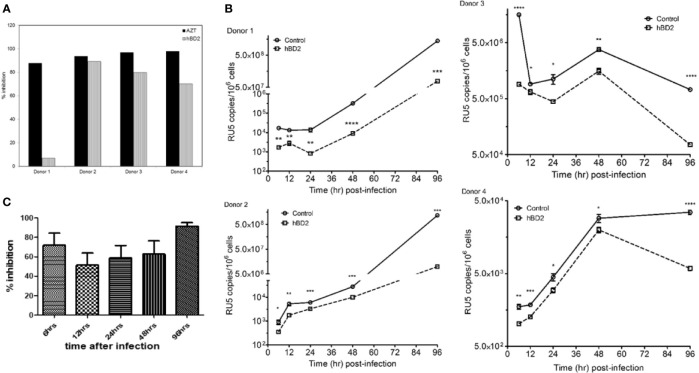
hBD2 suppresses HIV-1 at an early post-entry stage. **(A)** Single-cycle infection of MDM. Cells were infected with HIV-luciferase pseudotyped with AMLV envelope. After infection, cells were incubated 3 days in presence or absence of hBD2. Subsequently, cells were lysed and luciferase activity was measured. Percentage of inhibition was calculated for treated infected cells in reference to untreated infected cells. Data are presented for independent experiments from 4 different donors. **(B)** hBD2 inhibits accumulation of early reverse transcription products of HIV-1. MDM were challenged with DNase I-treated HIV-1_BaL_. Post-infection, hBD2 was added to the cultures. Total cellular DNA was isolated at the indicated time points and copies of LTR/RU5 products of reverse transcription were measured in triplicate by real-time PCR. Readings were averaged ± SEM and are presented as copies per million cells; log-scale graph. *P < 0.05, **P < 0.005, ***P < 0.0005, ****P < 0.0001 between treatment and control groups determined with unpaired two-tailed *t* test. Data are presented for independent experiments from 4 different donors and **(C)** summary graph of the average inhibition for the four donors shown in **3B** was determined as the percentage of HIV-1 DNA copies in treated infected cells in reference to untreated infected cells.

We and others have shown that hBD2 and hBD3 interact directly with HIV-1 decreasing infectivity irreversibly (Quinones-Mateu et al., 2003; Sun et al., 2005; Lafferty et al., 2010). In the current study, hBD2 was always added to macrophage cultures post-infection, decreasing the direct impact of hBD2 on HIV. To investigate whether hBD2 inhibits HIV-1 in macrophages post-entry, a single-cycle infection assay was employed with an HIV luciferase reporter virus pseudotyped with the AMLV envelope that does not use either CXCR4 or CCR5 for host entry. MDM were infected for 3 hours and incubated for 3 days in presence or absence of hBD2. AZT was used as a positive control. Subsequently, luciferase activity was measured and the percentage of inhibition compared with infected untreated cells was calculated. As shown in **Figure 3A**, hBD2 treatment resulted in significant inhibition (>70%) of luciferase expression in 3 of 4 donors tested. Treatment with AZT under the same conditions resulted in inhibitory activity greater than 80% in all donors. We observed donor-to-donor variability exemplified by the lack of inhibition with hBD2 in Donor 1 which is not unusual in studies with primary cells from a random pool of human subjects. This may be due to lack of hBD2 receptor(s) on the cell surface, or other parameters. Overall, these results show that hBD2-mediated restriction of HIV-1 is not dependent on the Env-host cell interaction, and occurs post-entry.

To probe the intracellular mechanism of inhibition, the presence of early (negative strand strong stop, -sss) products of HIV-1 reverse transcription were quantified in MDM infected with HIV-1_BaL_ by real-time quantitative PCR at various time points post-infection, using α-tubulin as the reference gene. Cells were infected, treated after infection with hBD2 (4.7 µM) and harvested for processing as described in *Materials and Methods*. For positive controls, cells were pretreated with either AZT or T20. Percent inhibition was calculated for ratio of HIV infected treated cells to infected untreated cells. The results in **Figures 3B, C** and **Figure S3** confirm that inhibition occurs post-entry, as HIV-1 DNA products are detected in the presence and absence of hBD2. After 6 hours post-infection, hBD2 treated samples showed significant inhibition of accumulation of LTR/RU5 products (**Figures 3C**, **S3**). Pretreatment with AZT also inhibited accrual of these products (data not shown). Some variability in the level of inhibition was observed in cells from different donors, ranging from 40-90%, but overall they all showed inhibition of early reverse transcription products. Thus, hBD2 restriction acts after the initiation of reverse transcription, preventing the completion of full length viral DNA products.

### hBD2 Inhibits HIV-1 in Macrophages Post-Entry *via* Gi-Protein Mediated Signaling

**Figure 4:**
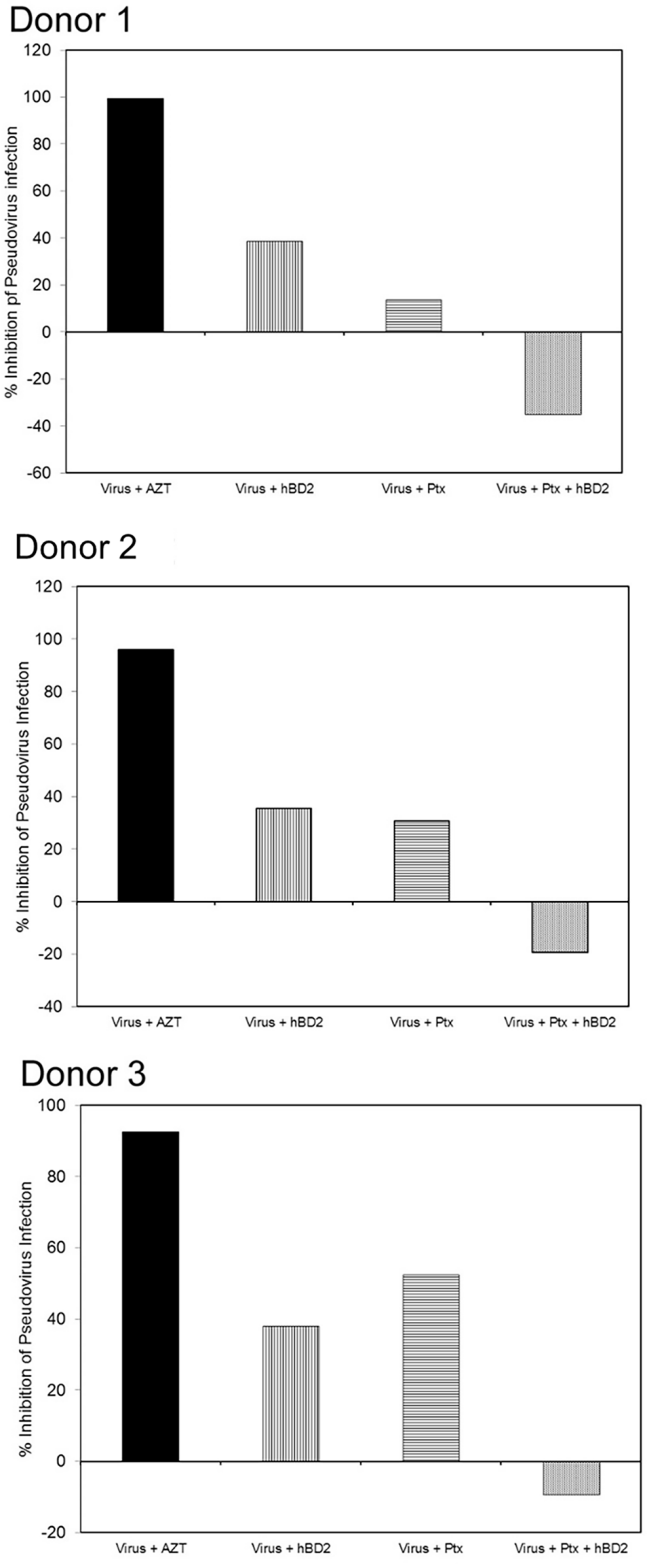
hBD2 inhibits HIV-1 in macrophages post-entry via G_i_-protein-mediated signaling. MDM were infected with single-cycle HIV-luciferase virus pseudotyped with AMLV envelope followed by pretreatment with or without PTx (100 ng/ml). Cells were then incubated 3 days in presence or absence of hBD2. Subsequently, cells were lysed and luciferase activity was measured. Percentage of inhibition was calculated for treated infected cells in reference to untreated infected cells. Data are presented for independent experiments from 3 different donors.

hBD2 is known to use C-C chemokine receptor 6 (CCR6) and C-C chemokine receptor 2 (CCR2) to induce cell migration in immature DCs, memory T (Yang et al., 1999) and mast cells (Niyonsaba et al., 2002), respectively, although there is evidence that additional receptors might be involved in the migration of cells of myeloid origin (Soruri et al., 2007). Preincubation of cells with pertussis toxin (PTx) abrogated migration towards hBD2, indicating that hBD2 signals *via* a receptor(s) coupled to PTx-sensitive G_αi_ proteins (Soruri et al., 2007). To elucidate whether the inhibition of HIV by hBD2 in macrophages is mediated by G_αi_-proteins signaling, we used PTx in our assays. MDM were infected with the AMLV pseudotyped HIV luciferase reporter virus and pretreated with PTx (100 ng/ml) and incubated for 3 days in presence or absence of hBD2. AZT was used as a positive control. Treatment of infections with PTx alone resulted in some inhibition of HIV LTR driven luciferase gene expression. Both inhibition (Alfano et al., 1999; Copeland et al., 1999; Alfano et al., 2000; Alfano et al., 2001; Iordanskiy et al., 2002; Lapenta et al., 2005; Hu et al., 2006) and enhancement (Momoi et al., 2000) of HIV infection in response to varying concentrations of PTx have been previously reported by different groups. However, in our experiments pretreatment with PTx resulted in abrogation of hBD2 inhibition of HIV (**Figure 4**), although inhibition was not completely reversed in all donors tested, which is indicative of more than one mechanism for hBD2 inhibition of HIV in macrophages. This result suggests that, at least in part, hBD2 binds GPCRs on the surface of macrophages and activates intracellular G_i_-protein signaling pathways to mediate its HIV inhibitory activity.

### Expression Pattern of Known hBD2 Receptors on the Surface of Macrophages

CCR6 is responsible for both hBD1 and hBD2 binding and chemotaxis of memory T cells, immature DCs (Yang et al., 1999), and TNF-α-treated neutrophils (Niyonsaba et al., 2004). Several studies have reported that hBD3 and hBD4 are chemotactic for peripheral blood monocytes (Wu et al., 2003; Soruri et al., 2007) and mast cells which do not express CCR6 (Soruri et al., 2007) implying the existence of an unidentified receptor. Two independent studies implicated CCR2 as the GPCR responsible for hBD2 and hBD3-mediated signaling and chemotaxis of monocytes (Jin et al., 2010; Rohrl et al., 2010). CCR2 is largely expressed on myeloid cells, such as monocytes (Katschke et al., 2001), DCs, macrophage subsets (Lin et al., 2008), and neutrophils (Iida et al., 2005).

**Figure 5:**
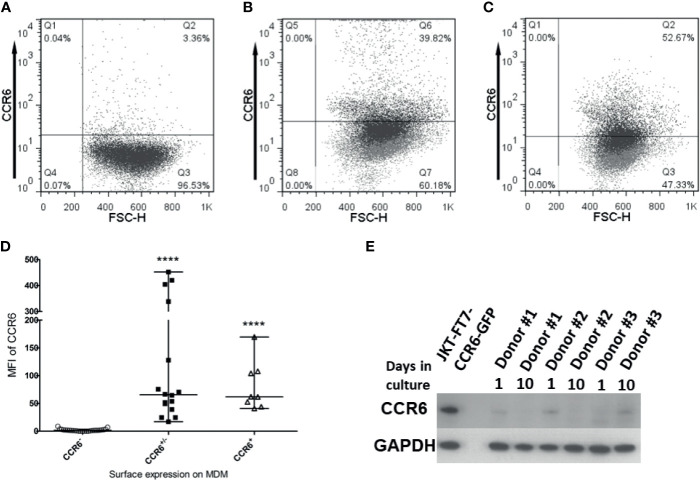
CCR6 is expressed on macrophages. Untreated, uninfected MDM were harvested and stained for flow cytometry analysis of CCR6 as described in *Materials and Methods*. Data analyses were performed using FlowJo software. Forward scatter dot plots show the fluorescence and percentage of cells positive for CCR6 as compared to the respective isotype-matched control. A representative of each type **(A)** CCR6^-^, **(B)** CCR6^+/-^, and **(C)** CCR6^+^ is shown. **(D)** Median Fluorescence Intensity (MFI) values for CCR6 surface expression (as compared to MFI for isotype control) on MDM from different donors designated as either CCR6^-^, CCR6^+/-^, or CCR6^+^. Data are presented as median and range of MFI values. Each dot represents one donor. ****P < 0.0001 between CCR6^+/-^ and CCR6^-^ or CCR6^+^ and CCR6^-^ determined with unpaired two-tailed *t* test. **(E)** Immunoblot analysis of CCR6 on MDM from 3 donors, 1 day and 10 days of tissue culture without treatment. JKT-FT CCR6 GFP cell line lysate in first lane from left is used as a positive control; the second lane is empty; subsequent lanes are donors #1-3, at day 1 and day 10 of tissue culture.

In order to identify the receptor(s) used by hBD2 on macrophages for intracellular signaling that results in restriction of HIV-1 replication, we analyzed the surface expression patterns of the known hBD2 receptors i.e. CCR2 and CCR6 on seven to ten days old MDM from healthy human donors (*n*=53) by flow cytometry. Based on the percentage of cells expressing the CCR, MDM were classified as follows: ≥50% cells positive= + (moderate to high levels); 10-49% cells positive= +/- (low to moderate); <10% cells positive= - (negative). Results are summarized in **Tables 2**, **3** representative MDM of each type is shown in **Figures 5A–C**. **Figure 5D** shows the MFI of CCR6 signal, as compared to its respective isotype-matched control, on MDM from these different donors. As shown in **Table 2**, only 4% of the donors were CCR2^+^ and 9% were CCR2^+/-^ compared to 87% that were CCR2^-^. In contrast, 15% of the same donors were CCR6^+^ and 28% were CCR6^+/-^ while 57% of the donors were CCR6^-^. However, as shown in **Table 3**, MDM from 51% of the donors were negative for both CCR2 and CCR6. These data indicate that hBD2 may be using additional receptors on primary human MDM.

**Table 2:** Expression of chemokine receptors from blood donors.

CCR Phenotype	%of Donors
CCR2+	4
CCR2 +/-	9
CCR2-	87
CCR6+	15
CCR6 +/-	28
CCR6-	57

key: - = negative; + = 50% cells positive; +/- = 10-49% cells positive.

**Table 3:** Patterns of co-expression of chemokine receptors from blood donors.

CCR Phenotype	No. of Donors	%of Donors
CCR2+ CCR6+	4	8
CCR2- CCR6-	27	51
CCR2 OR CCR6	22	42
Total (n)	53

N, no. of donors.

Since this is the first report of CCR6 surface expression on macrophages, we used additional biochemical methods to confirm CCR6 protein expression in MDM. To this end, we performed immunoblot analysis for CCR6 on whole cell lysates of MDM and used JKT-FT7 cell line (CCR6^-^) and primary human CD4^+^ T cells that were sorted into CCR6^+^ and CCR6^-^ populations as controls. As shown in **Figure 5E**, MDM do synthesize CCR6 protein at levels sufficient to be detected in immunoblots.

### hBD2 Can Signal *via* More Than One Receptor on Macrophages to Inhibit HIV-1

**Figure 6:**
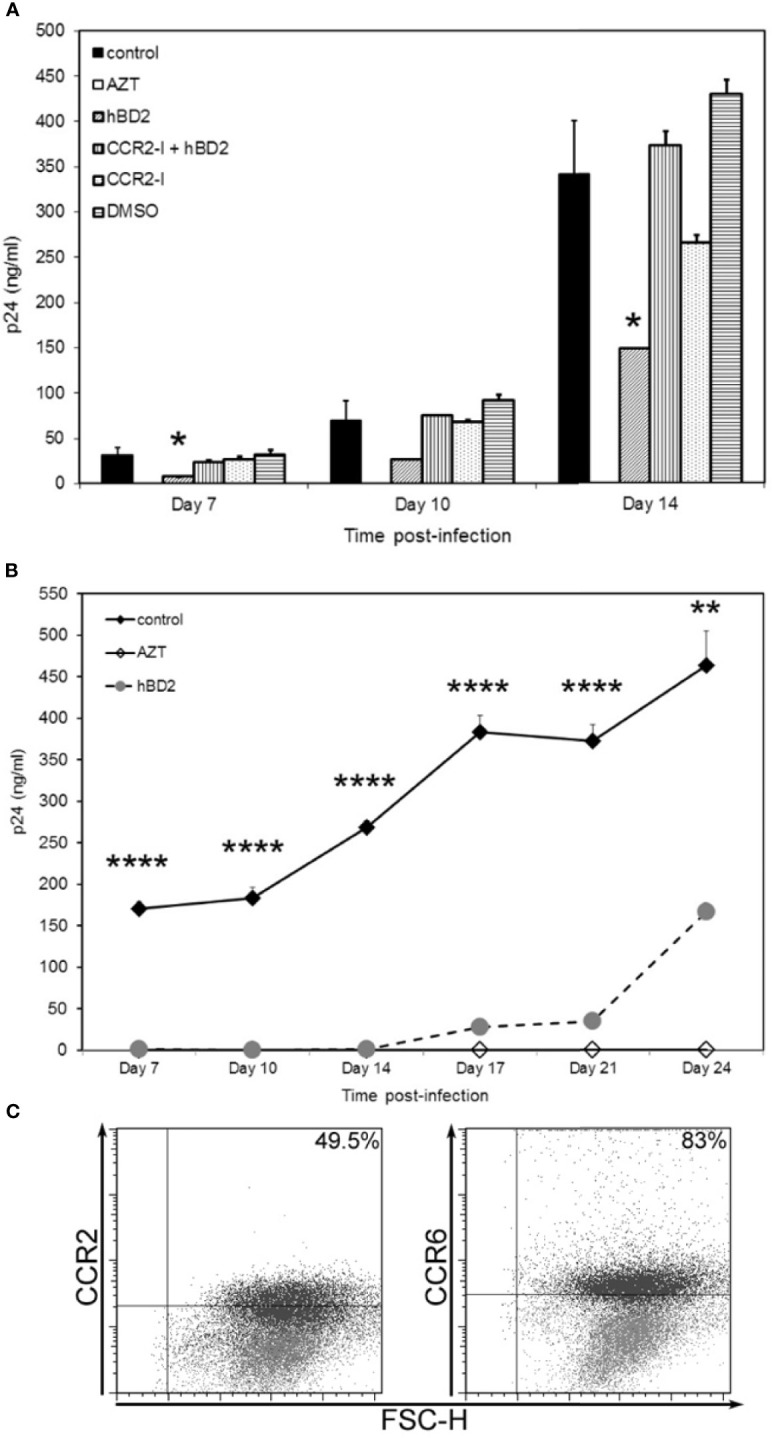
hBD2 can signal via more than one receptor type on macrophages. **(A)** Neutralization of CCR2 rescues HIV-1 infection. MDM were infected with HIV-1_BaL_. Cells were pretreated with AZT as control. Post-infection, infected untreated cells were pretreated with pharmacological antagonist RS102895 or DMSO control for 2 hrs followed by culture in presence or absence of hBD2 to the cultures. Infection was monitored by assaying supernatants for HIV p24 production by ELISA at the times indicated. Data are presented as mean ± SEM of triplicates. *P < 0.05 between treatment and control infection determined with unpaired two-tailed *t* test. Representative experiment, *n*=2. **(B)** hBD2 signals via an as yet unidentified receptor. MDM, that were CCR2- CCR6- (by FACS staining), prior to start of infection, were infected with HIV-1_BaL_. Cells were pretreated with AZT as control. Post-infection, cells were cultured in presence or absence of hBD2. Infection was monitored by p24 ELISA. Data are presented as mean ± SEM of triplicates. **P < 0.005, ****P < 0.0001 between treatment and control infection determined with unpaired two-tailed *t* test. **(C)** CCR2 and CCR6 surface expression levels vary with time. Uninfected, untreated cells from the donor used in **(B)** were harvested and stained for flow cytometry analysis as described in *Materials and Methods*. Data analyzed using FlowJo software. Forward scatter dot plots show the fluorescence and percentage of cells positive for CCR2 and CCR6 at Day 0 (grey) and Day 23 (black) as compared to the respective isotype-matched controls.

Although several studies show that CCR2 acts as the receptor for hBD2 and hBD3 on monocytes, Phillips et al. (Phillips et al., 2005) determined that CCR2 expression is down regulated as human monocytes gradually differentiate into macrophages. To determine whether CCR2, when expressed on MDM, is involved in hBD2-mediated intracellular inhibition of HIV-1, we used CCR2^+^ MDM. Cells were infected, pretreated with the potent and selective CCR2 pharmacological antagonist RS102895 (Jin et al., 2010) and subsequently cultured in the presence or absence of hBD2. As expected, hBD2 showed 60 to 75% inhibition of R5 virus replication over time in cells that were not treated with the inhibitor. The CCR2 antagonist, or DMSO control, by itself did not have a significant effect on infection. In contrast, blocking CCR2 resulted in complete reversal of HIV-1 inhibition by hBD2 (**Figure 6A**) suggesting a role for CCR2, when present, in mediating intracellular inhibition of the virus.

Since all MDM (from different donors) tested were not positive for surface expression of CCR2, CCR6 or both, we hypothesized that hBD2 signals in macrophages *via* additional receptors. To examine this possibility, we tested the ability of hBD2 to inhibit HIV-1 in CCR2^-^ CCR6^-^ MDM. Unexpectedly, we found that hBD2, at the same concentration used in previous infectivity experiments, completely abrogated HIV-1 replication in these cells up to 14 days post-infection, similar to the AZT control (**Figure 6B**). This is not attributable to low or no infection as all cells were infected in the same tube and split post-infection into infected untreated and infected hBD2-treated. These data suggest that hBD2 can use receptor(s) other than CCR2 and CCR6 on MDM. We decided to follow the infection over a longer time period, replenishing hBD2 in the culture every 3 days. As shown in **Figure 6B**, after 14 days, we observed virus replication gradually in the hBD2 treated cells, although inhibition (ranging from 92% to 64% on days 17 and 24, respectively) was still present. The MDM were also analyzed at the same time points for CCR2 and CCR6 surface expression. As seen in the scatter plots in **Figure 6C**, compared to no expression of either CCR2 or CCR6 at Day 0 (gray), 49% of the cells expressed CCR2 and 83% expressed CCR6 at Day 23 (black overlay on Day 0). Collectively, these data lead us to hypothesize that hBD2 uses different CCRs on macrophages with varying affinities.

### hBD2 Upregulates APOBEC3G and/or APOBEC3A in Macrophages

Our results suggest that the post-entry inhibition occurs during early reverse transcription. To explore the mechanism(s) by which hBD2 suppresses HIV-1, we examined its ability to affect host restriction factors, specifically members of the APOBEC3 family of cytidine deaminases, namely APOBEC3G (A3G) and APOBEC3A (A3A) which are known intracellular inhibitors of HIV-1 in macrophages (Dong et al., 2006; Peng et al., 2007; Hou et al., 2009; Thielen et al., 2010; Chaipan et al., 2013; Mashiba and Collins, 2013). In addition, data from our lab demonstrated that hBD2 induced A3G expression *via* CCR6 resulting in HIV inhibition in CD4^+^ T cells (Lafferty et al., 2010). Also, recent reports show that A3A expression is significantly upregulated and is the major cytidine deaminase in myeloid cells in response to IFN-α treatment (Peng et al., 2007; Koning et al., 2009; Thielen et al., 2010).

**Figure 7:**
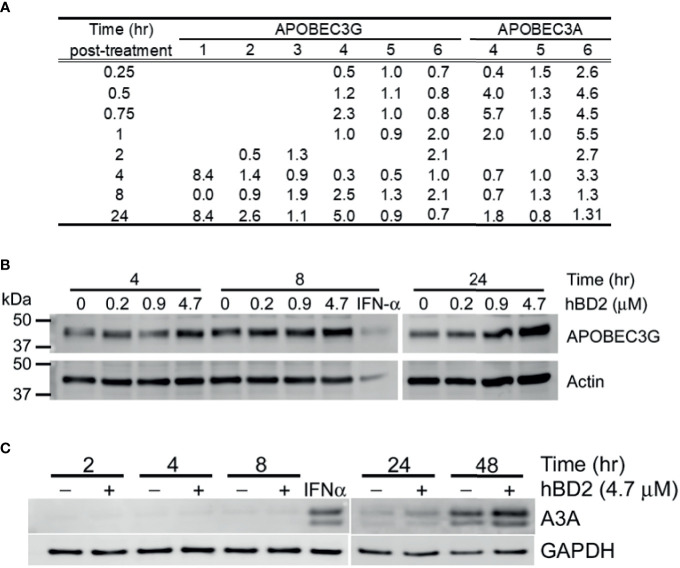
hBD2 upregulates APOBEC3G and/or APOBEC3A in macrophages. **(A)** APOBEC3G and APOBEC3A expression in response to hBD2. MDM were treated with hBD2 for indicated times and mRNA levels were assessed by quantitative real-time RT-PCR. The data was normalized to 18S ribosomal RNA. Triplicate measurements were used to calculate fold change as described in *Materials and Methods*. Data are presented as fold change in treated samples compared to untreated samples at matched time points. Data are for independent experiments from different donors. Analyses of APOBEC3G **(B)** and APOBEC3A **(C)** protein levels in response to hBD2. MDM were treated with hBD2 for various times and cell lysates were used to detect APOBEC3G and APOBEC3A proteins by western blotting. ß-actin and GAPDH serve as load controls. Representative experiment, n=3.

To determine whether hBD2 influences A3G and/or A3A expression, macrophages were treated with hBD2 and kinetics of message levels were determined by real-time RT-PCR. Treatment of cells with hBD2 induced a 2 to 3-fold increase in A3G mRNA signal in five of six donors tested, with one donor showing an 8-fold increase as compared to untreated cells, although we observed differences in the kinetics of induction among different donors (**Figure 7A**). In contrast, A3A levels increase from 1.5 to 6-fold within the first hour post-treatment with hBD2 compared with untreated cells, with variable kinetics between different donors (**Figure 7A**). To determine whether this increase in expression translated into increase in protein, cells were treated with hBD2 and lysates were prepared at 4, 8, and 24 hours. Equal amounts of total protein were subjected to immunoblotting with anti–human A3G antibodies. hBD2 enhanced A3G protein levels (**Figure 7B**) but high levels of endogenous A3G in untreated MDM made it difficult to decipher a clear increase in treated cell lysates from different donors. In case of A3A, we observed 1.5- to 2-fold increase in protein 24 to 48 hours post-treatment compared with endogenous levels in untreated MDM (**Figure 7C**). Peak protein signal was delayed compared to peak RNA signal for A3A, which may be due to differences in the kinetics of RNA and protein expression of this protein, and donor-to donor variability. Overall, gene expression and protein production analyses reveal that hBD2 induced both A3G and A3A in MDM with stronger induction of A3A.

## Discussion

We and others previously demonstrated that hBD2 and hBD3 inhibit HIV-1 replication in primary human PBMCs and CD4^+^ T cells (Quinones-Mateu et al., 2003; Sun et al., 2005; Lafferty et al., 2010; Lafferty et al., 2017). To the best of our knowledge, we demonstrate here for the first time that hBD2 and hBD3, but not hBD1, inhibit HIV-1 in human MDM. This inhibition occurs in the micromolar range of concentrations similarly to our previous observations on T cells, without effects on cell metabolism that we previously observed in CD4^+^ T cells treated with hBD3 (Sun et al., 2005). The dose-response profile of hBD2 that we observed was probably on primary cells was probably affected by donor-to-donor variability in expression of CCR6 and CCR2 (see below). The enhancement of HIV replication we observed by hBD1 was not observed in PBMC, where hBD1 has low inhibitory activity (Quinones-Mateu et al., 2003; Sun et al., 2005). The earlier studies on the effects of hBDs on HIV replication showed that the inhibitory activity was due to both a virucidal component, and an intracellular component (Sun et al., 2005). In this study, we addressed mostly intracellular mechanisms of inhibition in MDM. We further show that hBD2 acts *via* more than one receptor type to inhibit the virus post-entry, and not by altering surface expression levels of HIV receptor-co-receptors or by enhancing expression of anti-viral cytokines and β-chemokines. One mechanism is *via* binding to and signaling through different CCRs (GPCRs) on the cell surface. The known hBD2 receptor on monocytes, CCR2, is expressed on the surface of human MDM in 4% of donors we tested. We found CCR6 expression on MDM from more than 40% of the donors tested, albeit at varying levels. To our best knowledge, this is the first report of CCR6 expression on peripheral blood monocyte-derived macrophages. Our data differs from published data that CCR6 is not expressed on peripheral blood monocytes (Ruth et al., 2003; Rohrl et al., 2010) or macrophages (Soruri et al., 2007). However, low level of CCR6 expression has been reported on myeloid blasts from the peripheral blood of AML patients (Cignetti et al., 2003), and on CD14^+^ monocytes from peripheral blood and synovial fluid of rheumatoid arthritis patients (Ruth et al., 2003). Also, studies have reported surface expression of CCR6 on microglial cells (Flynn et al., 2003), the resident macrophages of the brain, astrocytes (Flynn et al., 2003), and spinal cord infiltrate macrophages (Mony et al., 2014). The broader biological significance of this discovery warrants further exploration because of the role of CCR6 in both pro- and anti-inflammatory immune responses. The lack of a good CCR6 specific neutralizing antibody, antagonist, or a human CCR6 expressing monocyte cell line at this time restricts our ability to test the role of CCR6 in hBD2-mediated inhibition of HIV in MDM, and/or whether some of the activity of hBD2 may be due to penetration of the peptide in cells.

Further support for our hypothesis that hBD2 signals in macrophages *via* more than one CCR comes from our experiments with PTx. MDM used from different donors for these experiments (**Figure 4**) expressed no CCR2 and either no CCR6 or low levels of CCR6 suggesting that these are not the only CCRs used by hBD2 on macrophages. Further, unexpectedly, maximum hBD2 inhibition of HIV was observed in MDM that were CCR2^-^ CCR6^-^ (**Figure 6B**). Therefore, we hypothesized that hBD2 may inhibit HIV in macrophages *via* additional receptors. This is consistent with a study which demonstrated that hBD2 mediates chemotaxis of mast cells *via* signaling through more than one receptor, and identified both high and low affinity receptors for hBD2 on this cell type (Niyonsaba et al., 2002). Our results on putative receptors for defensins open up more avenues of investigation as there may be more such β-defensin-CCR interactions on other cell types as well. Our results with PTx show that regardless of receptor usage, hBD2 requires Gαi signaling pathway(s) for HIV inhibition in macrophages. When Ptx was used in conjuction with hBD2 treatment, HIV replication was not just restored, but it appeared to be enhanced. While this finding does not invalidate the role of Gi-mediated signaling in the inhibitory effects of hBD2, It open the possibility that hBD2 may also induce activation of other intracellular pathways that increase HIV replication.

Similar to previous findings from our laboratory in CD4^+^ T cells (Lafferty et al., 2010; Lafferty et al., 2017), we found that post-entry hBD2 blocks virus replication at an early stage of the life cycle after the initiation of reverse transcription as evidenced by inhibition of the accumulation of early reverse transcription products. The intracellular inhibition is further mediated *via* upregulation of the innate anti-viral restriction factors APOBEC3G (A3G) and APOBEC3A (A3A) to different levels. We detected increased A3G RNA signal in most donors tested with a corresponding increase in protein levels at 24 hours in macrophage lysates. As mentioned in the *Results*, high signal for endogenous A3G detected in untreated MDM lysates complicates our ability to accurately determine the effect of hBD2 on the level of A3G protein synthesized in MDM. The level of variability we observed is not unexpected in primary cells, so that it is possible that the effects of hBD2 may vary *in vivo*. We observed A3A induction, although while RNA levels increased within the first hour following addition of hBD2, either very weak or no signal was observed for A3A protein in both untreated and treated cells until 48 hours post-treatment. This is similar to studies of IFN-α-induction of A3A in monocytes (Thielen et al., 2010) and macrophages (Goujon et al., 2013) that showed low levels of A3A protein 8-10 hours post-treatment, and more robust signal 24 hours post-treatment, hence, our results may be due to the kinetics of translation and/or the half-life of this protein in myeloid cells. Our data that hBD2 enhanced A3G and A3A expression support our model that elevated levels of APOBEC3 proteins contribute to hBD2-mediated anti-HIV-1 activity in macrophages.

Taken together, our results provide evidence that hBD2 has the ability to suppress HIV-1 infection of primary macrophages *in vitro*. While the role of macrophages in the initial stages of infection is still debated (Collins et al., 2000; Greenhead et al., 2000; Gupta et al., 2002; Hladik et al., 2007; Bouschbacher et al., 2008; Shen et al., 2011), it is widely accepted that macrophages are not only a major target of HIV during both the acute and chronic phases of disease-they produce and spread infectious virus-but also a major reservoir of latent virus that, especially in the CNS, eludes eradication by existing therapies. Since hBD2 is expressed by epithelial cells in mucosae and in the CNS by astrocytes (Hao et al., 2001), our findings could be relevant to both systemic infection and neurological complications of the disease. Indeed, a protective role for human ß-defensins against HIV acquisition in high risk exposed individuals has been suggested by Rugeles and colleagues (Zapata et al., 2008; Aguilar-Jimenez et al., 2013), and hBD2 has been shown to be correlated with ant- HIV activity in cervical-vaginal secretions (Ghosh et al., 2010; Patel et al., 2014). Several studies have also demonstrated an association between different ß-defensin gene polymorphisms and HIV infection in adults as well as children of diverse ethnicities (Braida et al., 2004; Milanese et al., 2006; Baroncelli et al., 2008; Milanese et al., 2009; Ricci et al., 2009; Aguilar-Jimenez et al., 2011; Freguja et al., 2012; Hardwick et al., 2012). Human ß-defensins, especially hBD2, therefore merit further consideration as both an immunotherapeutic and topical microbicide to prevent spread through sexual contact. While we did not address the effects of hBDs in a model of HIV-latency in MDM in this study, the possibility that hBDs can influence HIV reservoirs deserves to be addressed in further studies.

## Data Availability Statement

The datasets generated for this study are available on request to the corresponding author.

## Author Contributions

JB designed and performed experiments, wrote the article. LS performed experiment. WL contributed key reagents and edited the article. SG contributed key reagents, and edited the manuscript. AG-D conceived and supervised the study and edited the article. All authors contributed to the article and approved the submitted version.

## Funding

This work was supported by the National Institute of Neurological Disorders And Stroke award R01NS066842 to AG-D and the National Institute of Allergy and Infectious Diseases Research award R01AI061482 to WL. 

## Conflict of Interest

The authors declare that the research was conducted in the absence of any commercial or financial relationships that could be construed as a potential conflict of interest.
