# Supplementary material for: Human Beta-Defensin 2 and 3 Inhibit HIV-1 Replication in Macrophages
Source: Front Cell Infect Microbiol. 2021 Jul 1;11:535352. doi: 10.3389/fcimb.2021.535352 (PMC8281893; doi:10.3389/fcimb.2021.535352)
Supplement: Supplementary file 1 [file DataSheet_1.pdf]

A

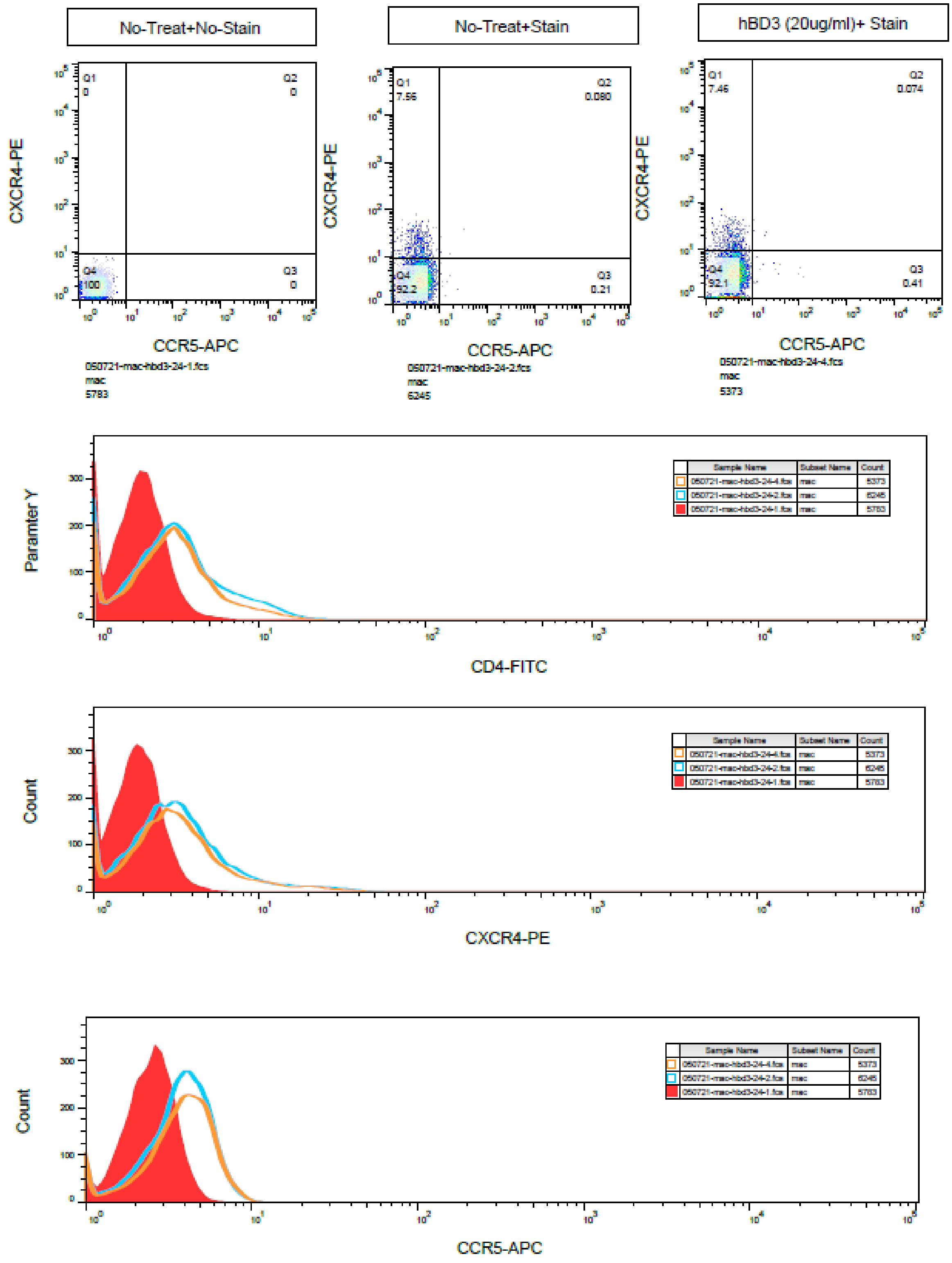

No-Treat+No-Stain
No-Treat+Stain
hBD3 (20ug/ml)+ Stain
Q1
0
Q2
0
Q3
0
Q4
100
Q1
7.56
Q2
0.080
Q3
0.21
Q4
92.2
Q1
7.46
Q2
0.074
Q3
0.41
Q4
92.1
CXCR4-PE
CCR5-APC
050721-mac-hbd3-24-1.fcs
mac
5783
050721-mac-hbd3-24-2.fcs
mac
6245
050721-mac-hbd3-24-4.fcs
mac
5373
Parameter Y
CD4-FITC
Count
CXCR4-PE
Count
CCR5-APC
Sample Name
Subset Name
Count

B

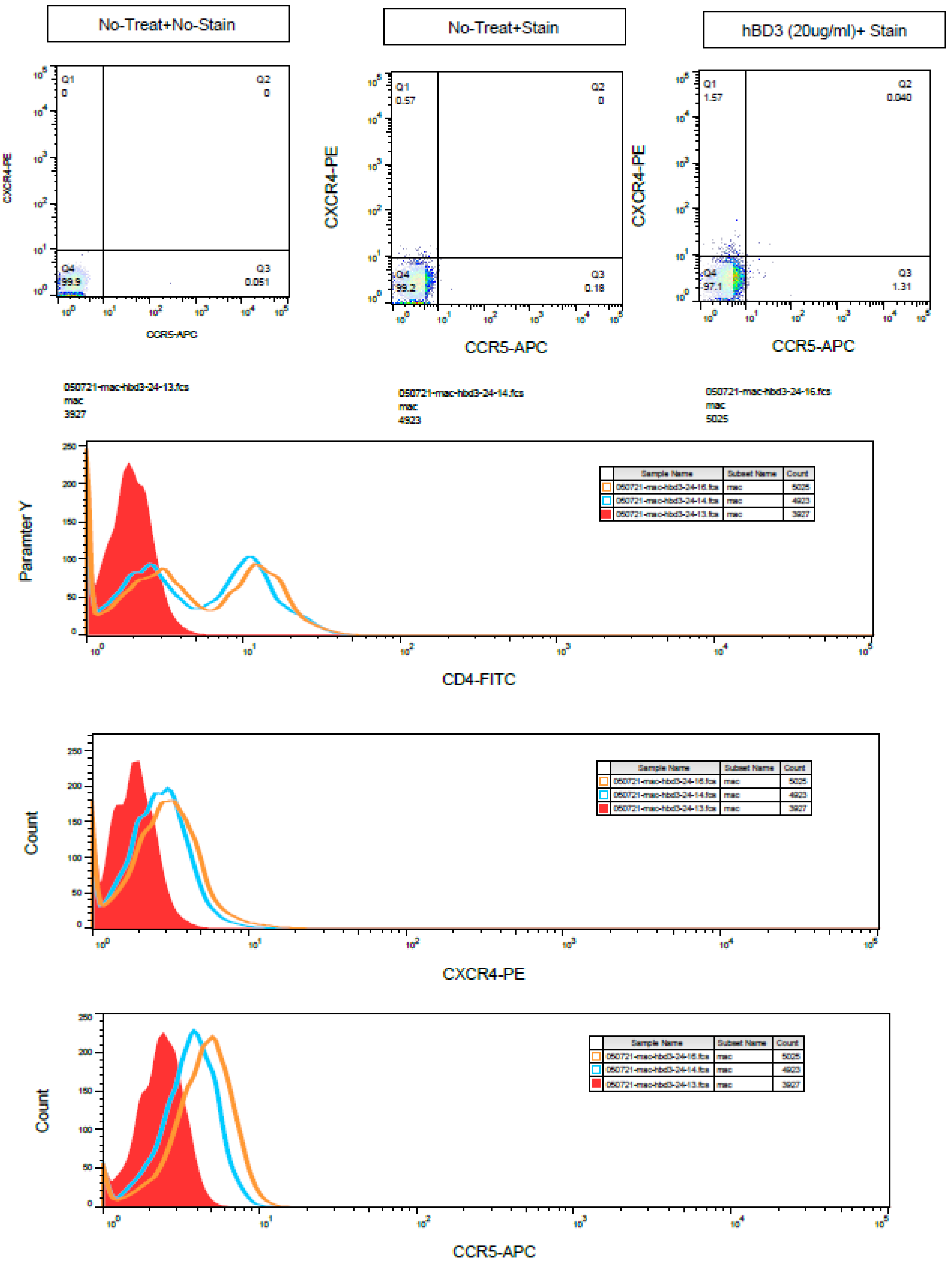
No-Treat+No-Stain
No-Treat+Stain
hBD3 (20ug/ml)+ Stain
Q1
0
Q2
0
Q4
99.9
Q3
0.051
CXCR4-PE
CCR5-APC
Q1
0.57
Q2
0
Q4
99.2
Q3
0.18
Q1
1.57
Q2
0.040
Q4
97.1
Q3
1.31
050721-mac-hbd3-24-13.fcs
mac
3927
050721-mac-hbd3-24-14.fcs
mac
4923
050721-mac-hbd3-24-16.fcs
mac
5025
Sample Name
Subset Name
Count
Parameter Y
CD4-FITC
Count
CXCR4-PE
Count
CCR5-APC

Supplemental Figure S1. hBD3 does not induce modulation of HIV receptors on the surface of MDM. MDMs were treated with hBD3 (4.7uM); 1, 2, 3 and 24 hours after treatment cells were analyzed for CD4, CCR5, CXCR4 expression by flow cytometry. Shown are the results of a representative experiment from one of 2 donors, 1 hour (A) and 24 hours (B) after treatment. In red, unstained cells. In blue, cells stained with the antibody as indicated below the graph; in orange, hBD2-treated cells.

A

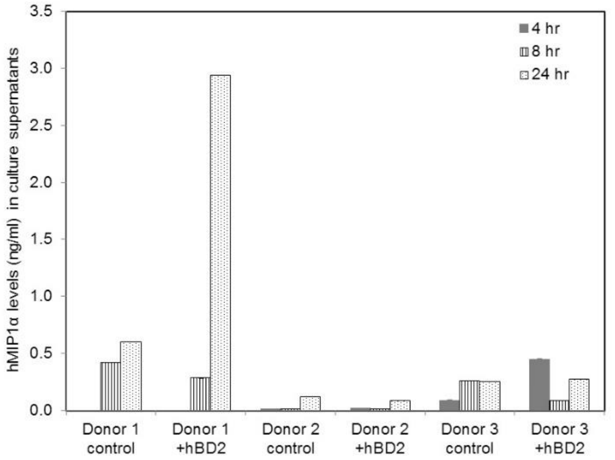

hMIP1α levels (ng/ml) in culture supernatants
3.5
3.0
2.5
2.0
1.5
1.0
0.5
0.0
4 hr
8 hr
24 hr
Donor 1 control
Donor 1 +hBD2
Donor 2 control
Donor 2 +hBD2
Donor 3 control
Donor 3 +hBD2

B

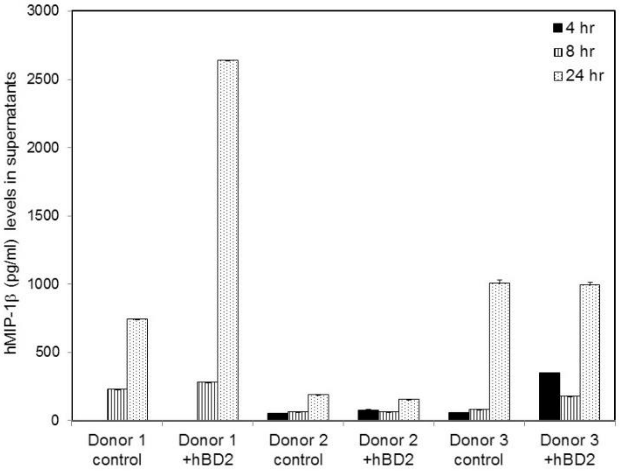

hMIP-1β (pg/ml) levels in supernatants
3000
2500
2000
1500
1000
500
0
4 hr
8 hr
24 hr
Donor 1 control
Donor 1 +hBD2
Donor 2 control
Donor 2 +hBD2
Donor 3 control
Donor 3 +hBD2

C

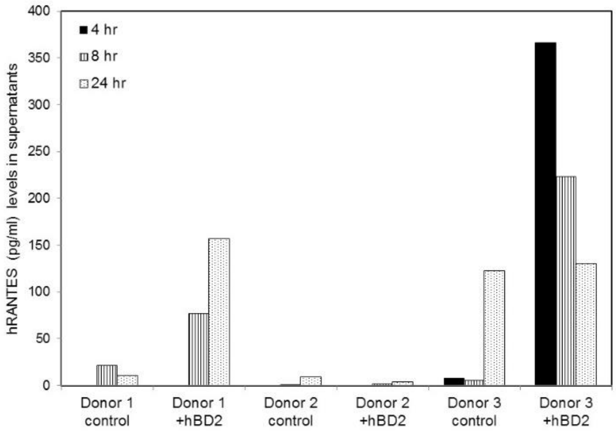

hRANTES (pg/ml) levels in supernatants
400
350
300
250
200
150
100
50
0
4 hr
8 hr
24 hr
Donor 1 control
Donor 1 +hBD2
Donor 2 control
Donor 2 +hBD2
Donor 3 control
Donor 3 +hBD2

Supplemental Figure S2. hBD2 does not induce expression of anti-viral cytokines or β-chemokines in macrophages. Culture supernatants from MDM treated with hBD2 were collected at times indicated and β-chemokines MIP-1α (A), MIP-1β (B), and RANTES (C) were quantified using commercial ELISA kits. Data are presented as average ± SEM of duplicates for independent experiments from 3 different donors.

1

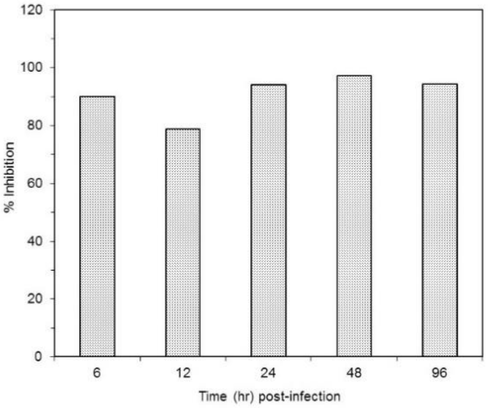
% Inhibition
Time (hr) post-infection
6
12
24
48
96
0
20
40
60
80
100
120

2

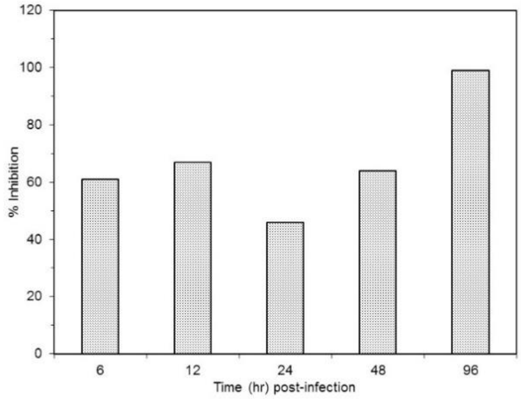
% Inhibition
Time (hr) post-infection
6
12
24
48
96
0
20
40
60
80
100
120

3

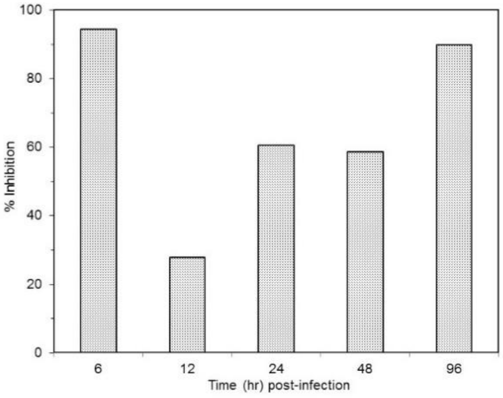
% Inhibition
Time (hr) post-infection
6
12
24
48
96
0
20
40
60
80
100

4

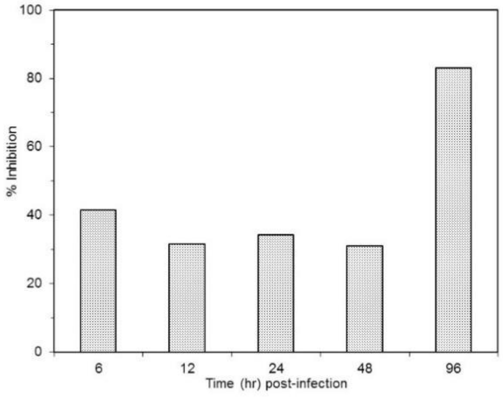
% Inhibition
Time (hr) post-infection
6
12
24
48
96
0
20
40
60
80
100

Supplemental Figure S3. hBD2 inhibits accumulation of early reverse transcription products of HIV-1. Inhibition for the four donors shown in Figure 3B was determined as the percentage of HIV-1 DNA copies in treated infected cells in reference to untreated infected cells.
